# IGF-1 and PDGF-bb Suppress IL-1β-Induced Cartilage Degradation through Down-Regulation of NF-κB Signaling: Involvement of Src/PI-3K/AKT Pathway

**DOI:** 10.1371/journal.pone.0028663

**Published:** 2011-12-14

**Authors:** Azadeh Montaseri, Franziska Busch, Ali Mobasheri, Constanze Buhrmann, Constance Aldinger, Jafar Soleimani Rad, Mehdi Shakibaei

**Affiliations:** 1 Department of Anatomical Sciences, Tabriz University of Medical Sciences, Tabriz, Iran; 2 Institute of Anatomy, Ludwig-Maximilian-University Munich, Munich, Germany; 3 Division of Veterinary Medicine, School of Veterinary Medicine and Science, Faculty of Medicine and Health Sciences, University of Nottingham, Sutton Bonington Campus, Sutton Bonington, United Kingdom; Technische Universität München, Germany

## Abstract

**Objective:**

Interleukin-1β (IL-1β) is a pro-inflammatory cytokine that plays a key role in the pathogenesis of osteoarthritis (OA). Growth factors (GFs) capable of antagonizing the catabolic actions of cytokines may have therapeutic potential in the treatment of OA. Herein, we investigated the potential synergistic effects of insulin-like growth factor (IGF-1) and platelet-derived growth factor (PDGF-bb) on different mechanisms participating in IL-1β-induced activation of nuclear transcription factor-κB (NF-κB) and apoptosis in chondrocytes.

**Methods:**

Primary chondrocytes were treated with IL-1β to induce dedifferentiation and co-treated with either IGF-1 or/and PDGF-bb and evaluated by immunoblotting and electron microscopy.

**Results:**

Pretreatment of chondrocytes with IGF-1 or/and PDGF-bb suppressed IL-1β-induced NF-κB activation via inhibition of IκB-α kinase. Inhibition of IκB-α kinase by GFs led to the suppression of IκB-α phosphorylation and degradation, p65 nuclear translocation and NF-κB-regulated gene products involved in inflammation and cartilage degradation (COX-2, MMPs) and apoptosis (caspase-3). GFs or BMS-345541 (specific inhibitor of the IKK) reversed the IL-1β-induced down-regulation of collagen type II, cartilage specific proteoglycans, β1-integrin, Shc, activated MAPKinase, Sox-9 and up-regulation of active caspase-3. Furthermore, the inhibitory effects of IGF-1 or/and PDGF-bb on IL-1β-induced NF-κB activation were sensitive to inhibitors of Src (PP1), PI-3K (wortmannin) and Akt (SH-5), suggesting that the pathway consisting of non-receptor tyrosine kinase (Src), phosphatidylinositol 3-kinase and protein kinase B must be involved in IL-1β signaling.

**Conclusion:**

The results presented suggest that IGF-1 and PDGF-bb are potent inhibitors of IL-1β-mediated activation of NF-κB and apoptosis in chondrocytes, may be mediated in part through suppression of Src/PI-3K/AKT pathway, which may contribute to their anti-inflammatory effects.

## Introduction

Osteoarthritis is a metabolically active, dynamic process that involves all joint tissues (cartilage, bone, synovium/capsule, ligaments and muscle). It is the most common clinical syndrome of joint pain accompanied by varying degrees of functional limitation and reduced quality of life [1]. Key pathological changes in OA include localised loss of articular cartilage and remodelling of adjacent bone with new bone formation (osteophyte) at the joint margins. These changes lead to episodes of chronic joint pain, limitation of movement and ultimately disability [2], [3]. At the molecular level OA is characterized by an imbalance between cartilage extracellular matrix (ECM) anabolism and catabolism which is mediated mainly by pro-inflammatory cytokines such as interleukin-1β (IL-1β) and tumor necrosis factor-α (TNF-α) [4]. IL-1β is one of the main cytokines that has been implicated in the pathogenesis of degenerative joint diseases such as OA and rheumatoid arthritis (RA) [5], [6]. This cytokine induces the releases of matrix degenerative enzymes (matrix metalloproteinases, MMPs) and inhibits the synthesis of extracellular matrix proteins in chondrocytes [7]. IL-1β also induces cell apoptosis, which leads to further degenerative changes in cartilage [8].

The remodeling of cartilage-specific matrix components is a pre-requisite for chondrocyte differentiation and survival [9]. β1-integrins are transmembrane signal transduction receptors in the chondrocyte membrane mediating essential cell–matrix interactions [10]. β1-integrins also regulate the interactions between chondrocytes and extracellular matrix macromolecules [10]. Disruption of cell-matrix interactions by inhibition of the MAPKinase pathway has been reported to lead to caspase-3 cleavage and chondrocyte apoptosis [11], [12]. Therefore, it is of great importance to elucidate the molecular and cellular mechanisms involved in cartilage inflammation and chondrocyte responses to pro-inflammatory cytokines in order to develop new treatments to protect cartilage in degenerative joint diseases.

Many pro-inflammatory effects of IL-1β and TNF-α in arthritis are regulated by activated ubiquitous central transcription factor nuclear factor-κB (NF-κB). In chondrocytes NF-κB is a key regulator of cyclooxygenase 2 (COX-2) and MMP expression [13], [14], [15], [16]. NF-κB regulates the expression of a large number of genes in response to infection, inflammation, adhesion, cell cycle and survival. In the absence of inflammatory signals NF-κB exists as an inactive cytoplasmic heterotrimer-complex by association with an inhibiting IκBα subunit. In response to phosphorylation, IκBα dissociates from the complex and the p65 and p50 subunits freely translocate to the nucleus and bind to NF-κB recognition sites in the promoter regions of various NF-κB regulated genes [17]. NF-κB appears to be a common target of multiple converging catabolic signalling pathways mediated by pro-inflammatory cytokines.

Pro-anabolic growth factors influence important cellular processes including differentiation, growth, survival and antagonize the effects of inflammatory mediators [18]. IGF-1 is one of the main anabolic growth factors in cartilage and plays an essential role in cartilage homeostasis and balancing proteoglycan synthesis. It stimulates proteoglycan and collagen type II production in chondrocytes [12], [19]. We have previously shown that IGF-1 plays an important role in chondrocyte differentiation; IGF-1 stimulation of the IGF-1 receptor activates key signaling proteins of the MAPK pathway [20]. The presence of PDGF-bb in cartilage defects exerts chemotactic and mitogenic effects on cells in the surrounding cartilage and could stimulate the infiltration of mesenchymal stem cells [4]. PDGF-bb also has a direct effect on chondrocyte proliferation, differentiation and cartilage proteoglycan production [21].

Although growth factors can prevent apoptosis by eliciting anti-apoptotic signals in chondrocytes [22] the mechanisms involved have not been elucidated at the molecular level. Despite the importance of PDGF-bb and IGF-1 as factors potentially capable of stimulating cartilage repair, very little is known about their anabolic effects on chondrocytes. Therefore the aim of this study was to investigate the hypothesis that PDGF-bb and IGF-1 can promote proliferation, differentiation and inhibit chondrocyte apoptosis in a synergistic manner by modulating the NF-κB pathway and associated signalling pathways and expression of downstream inflammatory molecules.

## Materials and Methods

### Antibodies

Antibodies against collagen type II (AB746), cartilage-specific proteoglycan (MAB 2015) and β1-integrin (MAB1977) and alkaline phosphatase linked sheep anti-mouse and sheep anti-rabbit secondary antibodies for immunoblotting were purchased from Millipore (Schwalbach, Germany). Antibodies to active caspase-3 (AF835), MMP-9 (MAB 911) and MMP-13 (MAB 511) were from R&D Systems, Inc., (Heidelberg, Germany). SOX-9 antibody was purchased from Acris Antibodies GmbH (Hiddenhausen, Germany). Cyclo-oxygenase-2 (160-112) antibody was obtained from Cayman Chemical (Ann Arbor, MI, USA). Monoclonal anti-β-actin was purchased from Sigma-Aldrich (Munich, Germany). Monoclonal anti-phospho-p42/p44 Erk1/2 and polyclonal anti-Shc were purchased from BD (BD Biosciences, Belgium). Antibodies against p65, pan-IκBα, phosphatidylinositol 3-kinase (PI-3K) p85 and c-Src were obtained from Santa Cruz Biotechnology (Santa Cruz, CA). Antibodies against phospho-specific IκBα (Ser 32/36) and anti-phospho-specific p65 (Ser536) were obtained from Cell Technology (Beverly, MA). Anti-IκBα kinase (IKK)-α and anti-IKK-β antibodies were obtained from Imgenex (Germany).

### Growth media and biochemicals

Growth medium (Ham's F-12/Dulbecco's modified Eagle's medium (50/50) containing 10% fetal calf serum (FCS), 25 µg/ml ascorbic acid, 50 IU/ml streptomycin, 50 IU/ml penicillin, 2.5 µg/ml amphotericin B, essential amino acids and L-glutamine) was obtained from Seromed (Munich, Germany). BMS-345541, Pronase, collagenase and Trypsin/EDTA (EC 3.4.21.4) were purchased from Sigma (Munich, Germany). Epon was obtained from Plano (Marburg, Germany). IGF-1, PDGF-bb and IL-1β were purchased from Acris (Hiddenhausen, Germany). Wortmannin and PP1 were purchased from Biomol (Plymouth Meeting, PA, USA). SH-5 was from Calbiochem (La Jolla, CA, USA).

### Chondrocyte isolation and culture

Cartilage tissue samples from healthy femoral head articular cartilage obtained during joint replacement surgery for femoral neck fractures were used to isolate primary canine chondrocytes as previously described in detail [23]. Samples were obtained during total hip replacement surgery at Clinic of Small Animals with fully-informed owner consent and ethical project approval from the ethical review committee of the Ludwig-Maximilian-University Munich, Germany. However, for our *in vitro* study on such materials no approval was necessary because no direct contact with live animals was involved at all.

Samples were obtained during total hip replacement surgery with fully-informed owner consent and ethical project approval from the ethical review committee of the Ludwig-Maximilian-University Munich, Germany. In total, the experiments were performed three times and samples from three different donors were used. Donor ages ranged from 5 to 7 years. Briefly, for chondrocyte isolation the cartilage sample was sliced into 1–2 mm thick slices and incubated first with pronase (2%/Hams-F12) (Roche Diagnostics), followed by collagenase incubation (0.2%/Ham's-F12) in a shaking water bath at 37°C. The digested sample was centrifuged at 1000 g/5 min and cells plated at 1×10^6^ cells per T75 flask at 37°C/5%CO_2_. The first medium change was performed after 24 h, and following medium changes three times per week. Chondrocytes were split at ca. 70% confluency and passaged twice.

### Experimental design

The experiments performed in this study were performed on primary chondrocytes and specifically designed to mimic cellular events that occur in the clinical condition of OA. To stimulate chondrocyte dedifferentiation processes in monolayer cultures, we adopted a model, which stimulates cells with the pro-inflammatory cytokine IL-1β (10 ng/ml). During monolayer expansion chondrocytes were cultured in whole cell culture medium containing 10% FCS. Chondrocytes were washed three times with serum-starved medium (containing only 0.5% FCS) and further incubated for 30 min with the same medium before initiating treatment with IL-1β. Serum-starved chondrocytes were stimulated with IL-1β alone or with IGF-1 (10 ng/ml), PDGF-bb (10 ng/ml), BMS-345541 (5 µM) or the combination of both growth factors (each 5 ng/ml) or pre-stimulated with IGF-1 (10 ng/ml), PDGF-bb (10 ng/ml), BMS (5 µM) or a combination of both growth factors (each 5 ng/ml) for 12 h before treating with IL-1β for an additional 24 or 48 h. The growth factor concentrations used in this study are the same as those in previous studies from our own laboratory and were calculated through dose dependent experiments on human articular chondrocytes. In a second approach, monolayer cultured chondrocytes, treated as above, were transferred to high-density cultures and cultured under identical conditions with serum starved-medium to examine the effects of IGF-1 and/or PDGF-bb on chondrocyte differentiation potential in a three dimensional environment. Three dimensional high-density culture was performed as previously described [24]. Briefly, 1×10^6^ cells were pipetted onto a nitrocellulose filter resting on a steel net bridge. Cell culture medium reached the filter medium interface and cells were nurtured through diffusion. After one day in culture cells aggregated and formed a pellet on the filter.

For investigation of NF-κB translocation and IκBα phosphorylation chondrocyte cultures were treated either with 10 ng/ml IL-1β or co-treated with 10 ng/ml IL-1β and IGF-1 (10 ng/ml) or PDGF-bb (10 ng/ml) or the combination of both growth factors (each 5 ng/ml) for 0, 5, 10, 20, 40 and 60 min and nuclear and cytoplasmic extracts were prepared. These experiments were performed in triplicate and the results are provided as mean values from three independent experiments.

### Isolation of chondrocyte cytoplasmic and nuclear extracts

Isolation of cytoplasmic and nuclear extracts was performed as previously described in detail [25]. Briefly, chondrocytes were trypsinized and washed twice in 1 ml ice cold PBS. The cell pellet was re-suspended in 400 µl hypotonic lysis buffer containing protease inhibitors and incubated on ice for 15 min. 12.5 µl of 10% NP-40 were added and the cell suspension vigorously mixed for 15 seconds. The extracts were centrifuged for 1.5 min. The supernatants (cytoplasmic extracts) were frozen at −70°C. Approximately 25 µl of ice cold nuclear extraction buffer was added to the pellets and incubated for 30 min with intermittent mixing. Extracts were centrifuged and the supernatant (nuclear extracts) transferred to pre-chilled tubes for storage at −70°C.

### Western blot analysis

For western blot analysis, monolayer cultures were treated on ice for 30 min with lysis buffer (50 mM Tris/HCl, pH 7.2/150 mM NaCl/(v/v) Triton X-100/1 mM sodium orthovanadate/50 mM sodium pyrophosphate/100 mM sodium fuoride/4 µg/ml pepstatin A/1 mM PMSF) as previously described [11]. After centrifugation at 9,000 g for 30 min, the supernatant was stored at −80°C until further use. Total protein content of each lysate was determined with the bicinchoninic acid system using BSA as standard (Uptima, Monlucon, France). Samples were reduced with 2-mercaptoethanol on a heat block for 10 min at 90°C and total protein concentrations were adjusted. Proteins were separated using SDS-PAGE under reducing conditions using 5%, 7.5%, 10% and 12% polyacrylamide gels and blotted onto nitrocellulose membranes using a transblot electrophoresis apparatus (Bio-Rad, Germany) for 60 min at 120 volts. Further, membranes were transferred to blocking buffer (skimmed milk powder in 1% PBS and Tween 20) for 2 h, and then incubated with primary antibody overnight at 4°C. Following washing 3×10 min with blocking buffer, membranes were incubated with secondary antibody for 90 min. After more washing in 0.1 M Tris buffer (0.05 M MgCl_2_/0.1 M NaCl), specific antigen-antibody complexes were detected by nitroblue tetrazolium and 5-bromo-4-chloro-3-indoylphosphate as substrates for alkaline phosphatase.

### Transmission electron microscopy

Cell cultures were fixed in Karnovsky's fixative for 30 min and post-fixed in a 1% OsO_4_ solution. Samples were dehydrated in an ascending alcohol series before they were embedded in Epon, and cut on a Reichert Ultracut. 2% uranyl acetate/lead citrate was used for contrasting these ultrathin sections, as previously described [12].

### Immune complex kinase assay

Immune complex kinase assay was performed as previously described in detail [23]. Briefly, to test the effect of BMS, IGF-1 or PDGF-bb on IL-1β-induced IKK activation, immune complex kinase assays were performed. The IKK complex was immunoprecipitated from whole chondrocyte lysates with antibodies against IKK-α and IKK-β and subsequently incubated with protein A/G-agarose beads (Pierce, Germany). After 2 h incubation, the beads were washed with lysis buffer and resuspended in a kinase assay solution containing 50 mM HEPES (pH 7.4), 20 mM MgCl_2_, 2 mM dithiothreitol, 10 µM unlabeled ATP and 2 mg substrate GST-IκBα (amino acid 1–54) and incubated at 30°C for 30 min. This was followed by boiling in SDS-PAGE sample buffer for 5 min. Proteins were separated using SDS-page under reducing conditions as described above. Phosphorylation of GST-IκBα was assessed using a specific antibody against phospho-specific IκBα (Ser 32/36). To demonstrate the total amounts of IKK-α and IKK-β in each sample, whole-cell proteins were separated using SDS-PAGE under reducing conditions as described above. Detection of IKK-α and IKK-β was performed by immunoblotting with either anti-IKK-α or anti-IKK-β antibodies.

### Pharmacological inhibition experiments

Chondrocytes (5×10^4^ cells/petri dish) were grown in growth medium for 24 h. PI-3K inhibitor (wortmannin), Src inhibitor (PP1) and Akt inhibitor (SH-5) treatments were carried out in serum-starved medium. Chondrocytes were pre-treated for 1 h with serum-starved medium containing wortmannin (20 nM), PP1 (10 µM) or SH-5 (10 µM), treated with IGF-1 (10 ng/ml) or PDGF-bb (10 ng/ml) or the combination of both growth factors (each 5 ng/ml) for 4 h, and then exposed to 10 ng/ml IL-1β for 1 h. After these treatments, nuclear extracts were prepared and examined for NF-κB as described in Materials and Methods.

### Statistical analysis

Specific binding of each protein was measured and semi-quantitatively analysed using the densitometric evaluation software package Quantity One (Bio-Rad Laboratories Inc., Munich, Germany). The results are shown as the mean ± SD of a representative experiment performed three times. Data shown are representative of three independent experiments.

## Results

To examine the effect of PDGF-bb or/and IGF-1 on the NF-κB activation pathway, we used IL-1β because the pathway activated by this cytokine is relatively well understood.

### IGF-1 or/and PDGF-bb inhibit IL-1β-induced degenerative features and apoptosis in chondrocytes in monolayer culture and promote redifferentiation of IL-1β-treated chondrocytes in high-density culture

**Figure 1:**
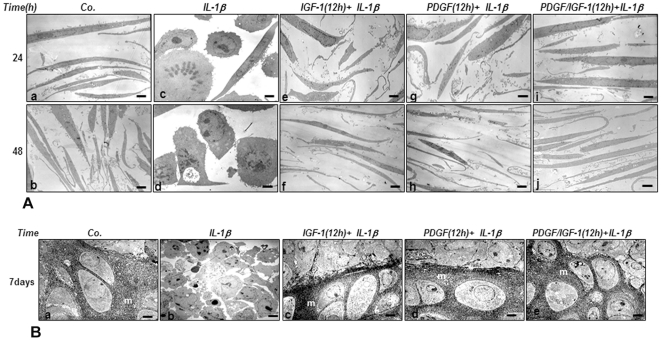
A: a–j: Effects of IGF-1 or/and PDGF-bb on IL-1β-induced apoptosis and cellular degeneration in chondrocytes. Transmission electron microscopy was performed to study the effects of IGF-1 or/and PDGF-bb on IL-1β-stimulated primary chondrocytes. Untreated control cultures consist of vital, active cells containing a well-developed rough endoplasmic reticulum, mitochondria and a well-organized cytoplasm after 24 or 48 h in culture (a and b). In contrast, stimulation with IL-1β for 24 h led to degenerative changes including condensation of heterochromatin, swelling of the rough endoplasmic reticulum and mitochondria (c). Longer exposure (48 h) to IL-1β resulted in more severe degenerative features including formation of apoptotic bodies and cell lysis (d). Pre-treatment of IL-1β-stimulated chondrocytes with IGF-1 or/and PDGF-bb for 24 or 48 h inhibited the degenerative effects of IL-1β (e–j). After 48 h (f, h and j), chondrocytes exhibited as large, viable and flattened cells with numerous tiny cytoplasmic processes, mitochondria, rough endoplasmic reticulum and other cytoplasmic organelles compared to control chondrocytes. **B: a–e: Redifferentiation of IL-1β-treated chondrocytes in high-density culture by IGF-1 or/and PDGF-bb.** Primary chondrocytes were either left untreated (a) or were treated with 10 ng/ml IL-1β (b), pre-treated with 10 ng/ml IGF-1 (c), 10 ng/ml PDGF-bb (d) or 5 ng/ml PDGF-bb and 5 ng/ml IGF-1 (e) for 12 h and then stimulated with IL-1β for another 24 h. The cells were transferred to high-density culture for seven days. Ultrastructural morphology was evaluated by electron microscopy. Control cultures showed characteristic features, including chondrocytes (c) embedded in a well-developed ECM (m) (**a**). Treatment with IL-1β for 24 h led to matrix breakdown and cell lysis (**b**). Pre-treatment with IGF-1 (**c**), PDGF-bb (**d**) or both growth factors in combination (**e**) resulted in a marked improvement of chondrocyte phenotype and the formation of cartilage nodules. The formation of a dense ECM (m) surrounding well-developed chondrocytes (c) was observed. ×5000; Bars: 1 µm.

We designed to investigate whether PDGF-bb or/and IGF-1 modulate degenerative effects of IL-1β in chondrocytes. Primary chondrocytes were exposed to indicated concentrations of PDGF-bb or/and IGF-1 before co-treatment with IL-1β as explained in materials and methods. The effect of PDGF-bb or/and IGF-1 on IL-1β-induced chondrocytes ultrastructural morphology and ECM formation was evaluated by electron microscopy. Untreated chondrocytes exhibited a typical fibroblast-like shape with mostly large euchromatic nuclei containing nucleoli, a well organized cytoplasm and tiny cytoplasmic processes after 24 and 48 h of culture (Fig. 1A: a and b). While treatment of chondrocytes with IL-1β, the cells altered their appearance, resulted in severe degenerative features such as swelling of rough endoplasmic reticulum and mitochondria, formation of vacuoles in cytoplasm and condensation of heterochromatin (Fig. 1A: c). Longer exposure (48 h) of chondrocytes to IL-1β led to more severe inflammation and cellular degeneration, such as formation of autophagic vacuoles in cytoplasm, lots of tiny cytoplasmic processes and also changes in flattened features of chondrocytes to rounded and apoptotic appearances (Fig. 1A: d). Chondrocytes that were pre-treated with IGF-1 or/and PDGF-bb for 12 h and then treated with IL-1β for an additional 24 or 48 h did not show severe ultrastructural and morphological changes (Fig. 1A: e–j). In the presence of growth factors, mitochondrial swelling, spherical chondrocytes and heterochromatin condensation disappeared (Fig. 1A: e–h). Co-treatment of chondrocytes with both growth factors decreased the characteristic signs of cellular destruction and organelle degeneration in a time dependent manner indicating a synergistic effect (Fig. 1A: i–j).

We have previously reported that high-density culture can promote chondrocyte differentiation since it supports cell-cell interactions [12], [26]. We examined whether PDGF-bb or/and IGF-1 can modulate IL-1β-induced dedifferentiation of chondrocytes in high-density culture. High-density cultures were stimulated with IL-1β alone or exposed to the indicated concentrations of growth factors before treatment with IL-1β. The ultrastuctural morphology of the cells and the formation of ECM macromolecules was evaluated using electron microscopy. Untreated chondrocytes were viable and exhibited round to oval shapes with large mostly euchromatic nuclei, mitochondria, rough endoplasmic reticulum and a well structured ECM (Fig. 1B: a). IL-1β resulted in degenerative and apoptotic features including formation of dense materials in nuclei, formation of blebs at the cell surface, formation of apoptotic bodies and degeneration of ECM structure (Fig. 1B: b). Stimulation of chondrocytes with PDGF-bb or/and IGF-1 before treatment with IL-1β resulted in more viable cells exhibiting round or oval shape, euchromatic nuclei with prominent nucleoli, a well-organized cytoplasm, lipid droplets and produced a well organized, dense and regular cartilaginous matrix (Fig. 1B: c–e). Taken together, PDGF-bb and IGF-1 in combination or alone reduced the degenerative and cytotoxic effects induced by IL-1β.

### Down-regulation of collagen type II, CSPG and β1-integrin-expression through IL-1β on chondrocytes is revoked by IGF-1 or/and PDGF-bb in monolayer cultures

**Figure 2:**
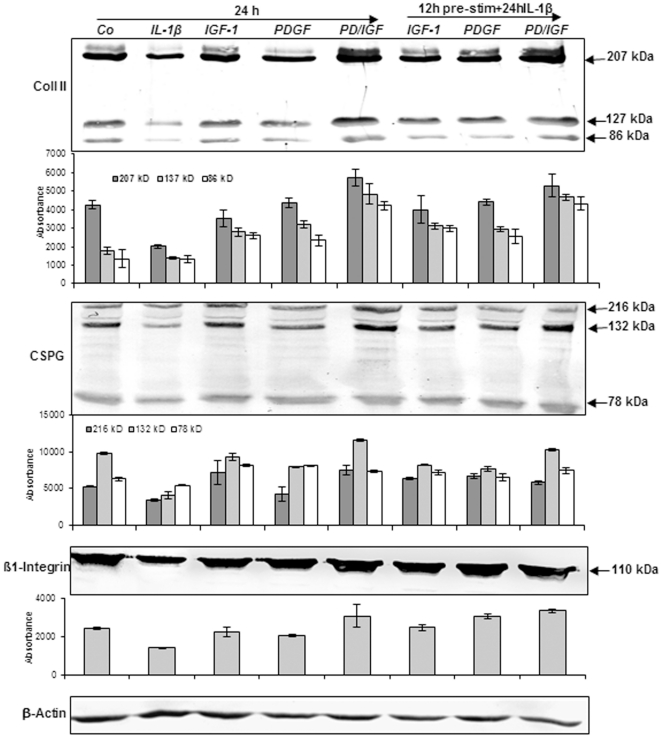
Effects of IGF-1 or/and PDGF-bb on IL-1β-induced inhibition of cartilage ECM expression in chondrocytes. Western blot analysis was performed to evaluate the effects of *IGF-1 or/and PDGF-bb* on IL-1β-induced inhibition of chondrogenic potential in chondrocytes. Whole cell lysates were probed with antibodies to collagen type II, cartilage specific proteoglycan (CSPG) and β1-integrin. Primary chondrocytes were treated with 10 ng/ml IL-1β, 10 ng/ml PDGF-bb, 10 ng/ml IGF-1 or 5 ng/ml PDGF-bb and 5 ng/ml IGF-1, or were pre-treated with 10 ng/ml PDGF-bb, 10 ng/ml IGF-1 or 5 ng/ml PDGF-bb and 5 ng/ml IGF-1 for 12 h and then stimulated with IL-1β for 24. Untreated chondrocytes had strong production of collagen type II, CSPG and β1-integrin, and stimulation with IL-1β alone markedly reduced these proteins. Pre-treatment of the chondrocytes with IGF-1 or/and PDGF-bb, however inhibited adverse effects of IL-1β. This was confirmed by quantitative densitometry. Expression of the housekeeping gene β-actin remained un-affected.

Immunoblotting of cartilage ECM components such as collagen type II, CSPG and the signaling and adhesion molecule β1-integrin was performed to confirm the results obtained using ultrastructural techniques (Fig. 2).

Collagen type II, CSPG and β1-integrin expression was down-regulated in chondrocytes stimulated with IL-1β after 24 (Fig. 2) and 48 h (data not shown). Chondrocytes treated with PDGF-bb or/and IGF-1 exhibited augmented collagen type II, CSPG and β1-integrin expression (Fig. 2) in comparison to IL-1β treated cells. Pre-treatment with PDGF-bb or/and IGF-1 followed by stimulation with IL-1β resulted in prevention of IL-1β induced effects on ECM production (Fig. 2). Co-treatment of chondrocytes with a combination of both growth factors increased the levels of these proteins compared with each treatment alone. Densitometric evaluation of a representative western blot experiment was performed in triplicate.

### IGF-1 or/and PDGF-bb suppress IL-1β-induced inhibition of Shc, Erk 1/2 and SOX-9 in chondrocytes in monolayer culture

**Figure 3:**
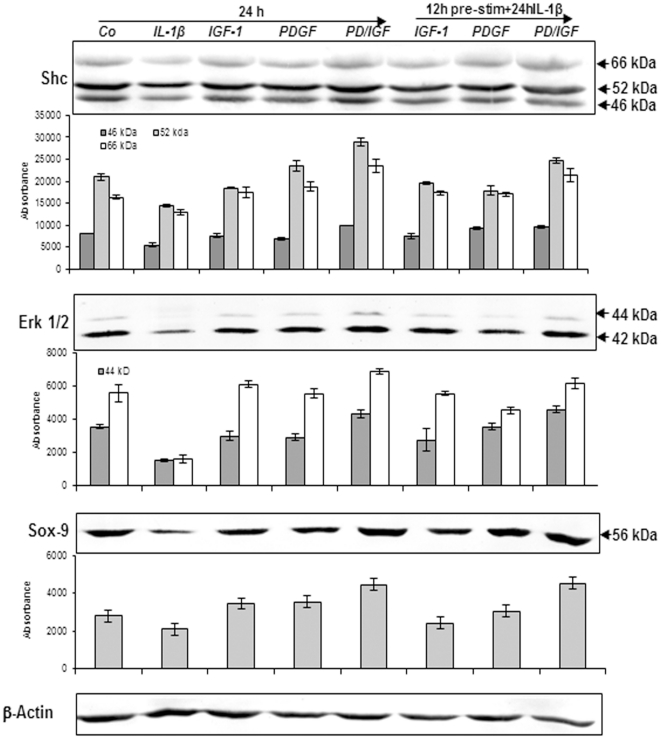
Effects of IGF-1 or/and PDGF-bb on IL-1β-induced inhibition of signaling proteins expression in chondrocytes. To evaluate the effects of IGF-1 and/or PDGF-bb on IL-1β-induced inhibition of MAPK signaling proteins in chondrocytes, whole cell lysates were probed with antibodies to Shc, Erk1/2 and SOX-9. Primary chondrocytes were either stimulated with 10 ng/ml IL-1β, 10 ng/ml PDGF-bb, 10 ng/ml IGF-1 or combination of both growth factor (5 ng/ml each) or pre-treated for 12 h with 10 ng/ml PDGF-bb, 10 ng/ml IGF-1 or combination of both (5 ng/ml each) followed by 10 ng/ml IL-1β for 24. Untreated cultures showed high expression of Shc, Erk1/2, and SOX-9, while IL-1β alone resulted in inhibition of Erk1/2, Shc as well as SOX-9 production. However, pre-treatment of cultures with IGF-1 or/and PDGF-bb inhibited the adverse effects of IL-1β and chondrocytes produced large amounts of Shc, Erk1/2 and SOX-9 at levels similar to control cultures. The results were confirmed by quantitative densitometry.

Extracellular signal regulated kinase1/2 (Erk1/2) and Shc regulate chondrocyte apoptosis, proliferation and also activities of several nuclear transcription factors, such as SOX-9 [27]. In this study we found that PDGF-bb or/and IGF-1 up-regulated the adaptor protein Shc (a more intense 52 kDa band in comparison to 66 and 46 kDa bands) and phosphorylated Erk1/2 (a more intense 42 kDa band in comparison to 44 kDa band) and inhibited down-regulation in Shc and phosphorylated Erk1/2 expression induced by IL-1β (Fig. 3). Interestingly, a combination of both growth factors exhibited stronger stimulation in Shc expression and Erk1/2 phosphorylation than each growth factor by itself (Fig. 3). The cartilage master transcription factor SOX-9 regulates cartilaginous specific matrix production and plays a pivotal role in chondrocyte differentiation [28]. To investigate whether growth factors are potent to activate SOX-9 in chondrocytes, primary chondrocytes in monolayer culture were either left unstimulated or stimulated with PDGF-bb or/and IGF-1 or were pretreated with PDGF-bb or/and IGF-1 for 12 h and then stimulated with IL-1β for 24 h (Fig. 3) and 48 h (data not shown), and the cell lysates were analyzed by immunoblotting. The results demonstrated that PDGF-bb or/and IGF-1 stimulated SOX-9 expression and inhibited IL-1β-induced decreases in SOX-9 expression. Quantitative analysis of western blot results performed in triplicate (Fig. 3) exhibited treatment of chondrocytes with PDGF-bb or/and IGF-1 stimulated SOX-9 expression in comparison to IL-1β treated chondrocytes (Fig. 3).

Taken together, these results showed that PDGF-bb or/and IGF-1 augment Shc, Erk1/2 and SOX-9 expression and counteract IL-1β-induced effects on the above-mentioned molecules in chondrocytes (Fig. 3).

### IGF-1 or/and PDGF-bb inhibit IL-1β-induced NF-κB dependent pro-inflammatory and matrix degradation and apoptotic gene products in chondrocytes in monolayer cultures

We investigated whether PDGF-bb or/and IGF-1 can modulate IL-1β-induced NF-κB-regulated gene products involved in the inflammation and degradation processes in cartilage tissue. It has been already implicated that IL-1β stimulation activates COX-2, MMP-9, MMP-13 and caspase-3 cleavage [13], [16].

**Figure 4:**
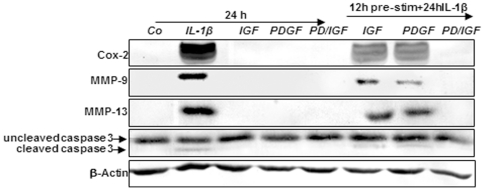
Effects of IGF-1 or/and PDGF-bb on IL-1β-induced NF-κB-dependent pro-inflammatory, pro-apoptotic and matrix degrading gene products in chondrocytes. To determine whether *IGF-1* or/and *PDGF-bb*exert effects on IL-1β-induced NF-κB-dependent expression of pro-inflammatory, pro-apoptotic and matrix degrading gene products, primary chondrocytes were either stimulated with 10 ng/ml IL-1β, 10 ng/ml PDGF-bb, 10 ng/ml IGF-1 or combination of both growth factors (5 ng/ml each) or pre-stimulated for 12 h with 10 ng/ml PDGF-bb, 10 ng/ml IGF-1 or combination of both growth factors (5 ng/ml each) followed by 10 ng/ml IL-1β for 24. Equal amounts of total proteins were separated by SDS-PAGE and analyzed by immunoblotting using antibodies raised against COX-2, MMP-9 and MMP-13 and active caspase-3. Stimulation with IL-1β resulted in production of COX-2, MMP-9, MMP-13 and caspase-3 cleavage. Pre-treatment with a combination of both IGF-1 or/and PDGF-bb downregulated COX-2, MMP-9, MMP-13 and cleaved caspase-3.

These experiments were designed to determine whether both growth factors are able to inhibit expression of proteins activated by IL-1β. Expression of COX-2, MMP-9, MMP-13 and caspase-3 cleavage was prominent in chondrocytes stimulated with IL-1β and was blocked in chondrocytes treated with PDGF-bb or/and IGF-1 (Fig. 4). Expression of above mentioned proteins were not inhibited completely by each growth factor alone in IL-1β-stimulated cells, whereas combinational pre-treatment of the growth factors was effective in inhibition of these pro-inflammatory and matrix degrading enzymes in the same manner in chondrocytes (Fig. 4).

### Cumulative inhibition effect of IGF-1 or/and PDGF-bb on IL-1β-induced NF-κB activation in chondrocytes in monolayer cultures

**Figure 5:**
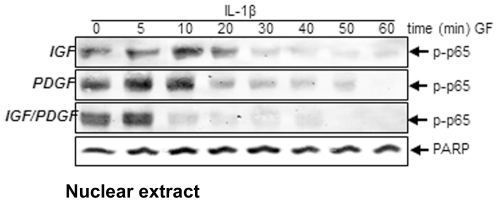
Inhibition of IL-1β-induced NF-κB activation and nuclear translocation by IGF-1 or/and PDGF-bb in primary chondrocytes. Primary chondrocytes were either stimulated with 10 ng/ml IL-1β, pre-stimulated with 10 ng/ml PDGF-bb, 10 ng/ml IGF-1 or a combination of both growth factors (5 ng/ml each) for the indicated times and then treated with 10 ng/ml IL-1β for 30 min. Nuclear extracts were prepared and assayed for NF-κB activation by western blot analysis. The results shown are representative of three independent experiments.

Based on the above data, we speculated that these growth factors might inhibit the NF-κB pathway. NF-κB is a regulator of inflammatory cytokines gene expression and plays a critical role in inflammatory responses. After phosphorylation, ubiquitination and degradation of Iκ-Bα, NF-κB translocates to the nucleus and binds (thus activating) the promoter of target genes [17]. To evaluate, whether IGF-1 or/and PDGF-bb inhibit the IL-1β-induced activation of NF-κB, nuclear protein extracts from serum-starved chondrocytes were probed for the phosphorylated form of NF-κB after pre-treatment with IGF-1, PDGF-bb and with IGF-1 and PDGF-bb for the indicated times followed by IL-1β stimulation for 30 min (Fig. 5). The western blot analysis confirmed that co-treatment of IGF-1 or PDGF-bb inhibited IL-1β-induced NF-κB activation in a time-dependent manner. Moreover, addition of both growth factors showed synergistic effects and inhibited the IL-1β-induced NF-κB activation. Taken together, these results indicate that IGF-1 and PDGF-bb act synergistically in inhibiting the activation of NF-κB more than each other.

### BMS-345541 (specific IKK inhibitor) suppresses IL-1β-induced inhibition of cartilage specific matrix, Shc, Erk 1/2 and SOX-9 in chondrocytes in monolayer culture

**Figure 6:**
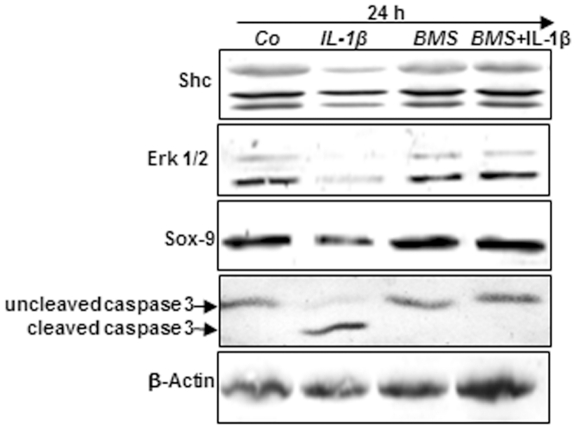
IKK inhibitor suppresses IL-1β-induced inhibition of Shc, Erk 1/2, SOX-9 and stimulation of caspase-3 cleavage in chondrocytes in monolayer culture. Primary chondrocytes were either stimulated with 10 ng/ml IL-1β, 5 µM BMS-345541 (BMS) or were pre-stimulated for 12 h with 5 µM BMS followed by 10 ng/ml IL-1β for 24. Whole cell lysates were probed with antibodies to Shc, Erk1/2, SOX-9 and active caspase-3. Untreated cultures showed high expression of Shc, Erk1/2 SOX-9 and no cleavage of caspase-3, while incubation with IL-1β alone resulted in suppression of Shc, Erk1/2 as well as SOX-9 production and stimulation of caspase-3 cleavage. However, pre-treatment of cultures with BMS inhibited the adverse effects of IL-1β and chondrocytes produced large amounts of Shc, Erk1/2 as well as SOX-9 proteins and the production of active caspase-3 was inhibited.

We next focused on the causal relationship between growth factor receptors and NF-κB signaling pathways. BMS is a potent and specific IKK inhibitor and can effectively inhibit NF-κB activation induced by diverse stimuli [29], [30]. Therefore, we treated primary chondrocytes with BMS to determine whether the effects of IL-1β (as well as their reversal by IGF-1 and PDGF-bb) on chondrocytes is also associated with an altered activation status of the IKK-NF-κB pathway and whether it suppressed the destructive effects of IL-1β in a similar manner to IGF-1/PDGF-bb. Serum starved chondrocytes were treated with BMS or IL-1β, or pre-treated with BMS and then co-treated with IL-1β. As shown in Figure 6, treatment of chondrocytes with IL-1β alone inhibited cartilage specific matrix expression (data not shown), phosphorylated Erk1/2, Shc, SOX-9 expression and stimulated caspase-3 cleavage (Fig. 6). The basal levels of cartilage specific matrix expression (data not shown), phosphorylated Erk1/2, Shc, SOX-9 expression and caspase-3 cleavage were not significantly changed after incubation with BMS (Fig. 6). Moreover, chondrocytes exposed to 5 µM BMS for 12 h followed by stimulation with IL-1β for 24 h, still exhibited a significant increase in cartilage specific matrix expression (data not shown), Erk1/2 phosphorylation, Shc, SOX-9 expression and exhibited a significant decrease in caspase-3 cleavage in a similar way to PDGF-bb or/and IGF-1 (Fig. 6). Taken together, these findings demonstrate that the IKK inhibitor (BMS) suppresses, in a similar way to PDGF-bb or/and IGF-1, the destructive effects of IL-1β and that IKK (at least in part) is one the kinases which plays an important role in growth factor receptor signalling pathways in chondrocytes.

### IGF-1 or/and PDGF-bb inhibit IL-1β-dependent IκBα degradation in chondrocytes in monolayer cultures

**Figure 7:**
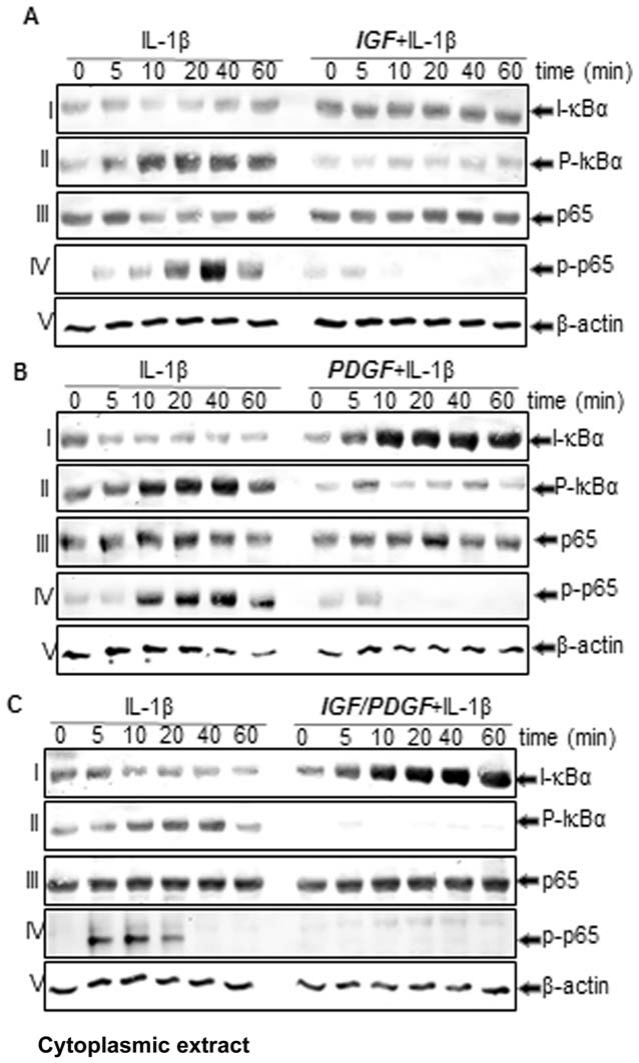
A–C: Effects of IGF-1 or/and PDGF-bb on IL-1β-induced IκB-α phosphorylation and degradation and p65 phosphorylation in chondrocytes in monolayer cultures. Primary chondrocytes were either stimulated with 10 ng/ml IL-1β, pre-stimulated with 10 ng/ml PDGF-bb, 10 ng/ml IGF-1 or a combination of both growth factors (5 ng/ml each) for 12 h and treated with 10 ng/ml IL-1β for the indicated times. Cytoplasmic extracts were prepared, fractionated (500 ng protein per lane) on 10% SDS–PAGE, and electrotransferred onto nitrocellulose membranes. Western blot analysis was performed with anti-phospho-specific-IκB-α, anti-IκB-α, anti-phospho-specific p65, anti-p65 antibodies and anti-β-actin (control). The results shown are representative of three independent experiments.

PDGF-bb and IGF-1 inhibited IL-1β-induced activation of NF-κB. Therefore, we examined the upstream mechanisms of NF-κB activation by IL-1β in chondrocytes. It is well known that the phosphorylation and degradation of IκBα, the natural blocker of NF-κB is required for the activation of NF-κB [31]. To test whether inhibition of IL-1β-induced NF-κB activation occurs through inhibition of IκBα degradation, we treated cells with PDGF-bb and IGF-1, followed by IL-1β stimulation and probed them for NF-κB activation in the nucleus and for IκBα activation in the cytoplasm by western blot analysis. The activation of NF-κB increased in the presence of IL-1β, whereas PDGF-bb or IGF-1-treated cells showed significantly decreased NF-κB activation even after 60 min of IL-1β stimulation (Fig. 7A–B). Interestingly, co-treatment of the chondrocytes with combinations of the two growth factors completely suppressed the IL-1β-induced NF-κB activation (Fig. 7C). IL-1β induced IκBα degradation in control cells within 5 min but not at all in PDGF-bb or/and IGF-1-treated cells (Fig. 7A–C). These results indicated that PDGF-bb and IGF-1 inhibit both IL-1β-induce NF-κB activation and IκBα degradation. Data shown are representative of three independent experiments.

### IGF-1 or/and PDGF-bb block IL-1β-dependent IκBα Phosphorylation in chondrocytes in monolayer cultures

To examine whether the inhibition of IL-1β-induced IκBα degradation is through inhibition of IκBα phosphorylation, chondrocytes were treated with PDGF-bb or/and IGF-1 and then with IL-1β and examined for IκBα phosphorylation in the cytoplasm by western blot analysis. IL-1β-induced IκBα phosphorylation was almost completely blocked by PDGF-bb or IGF-1 (Fig. 7A–B). Interestingly, co-treatment of the chondrocytes with combinations of the two growth factors completely suppressed the IL-1β-induced IκBα phosphorylation (Fig. 7C).

### IGF-1 or/and PDGF-bb block IL-1β-induced phosphorylation and nuclear translocation of NF-κB (p65) in chondrocytes in monolayer cultures

**Figure 8:**
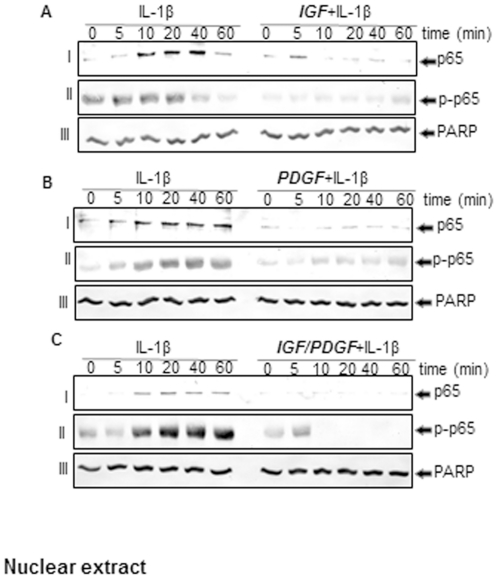
A–C: Effects of IGF-1 or/and PDGF-bb on IL-1β-induced p65 phosphorylation and nuclear translocation in chondrocytes in monolayer cultures. Primary chondrocytes were either stimulated with 10 ng/ml IL-1β, pre-stimulated with 10 ng/ml PDGF-bb, 10 ng/ml IGF-1 or combination of both growth factors (5 ng/ml each) for 12 h and treated with 10 ng/ml IL-1β for the indicated times. Nuclear extracts were prepared, fractionated (500 ng protein per lane) on 10% SDS–PAGE and electrotransferred onto nitrocellulose membranes. Western blot analysis was performed with anti-phospho-specific-p65, anti-p65 antibodies and anti-PARP (control). The results shown are representative of three independent experiments.

Western blot analysis showed that IL-1β induced the phosphorylation of the cytoplasmic pool of p65 in a time-dependent manner, and p65 phosphorylation could be seen as early as 5 min (Fig. 8). Pre-treatment with PDGF-bb or/and IGF-1 inhibited the IL-1β-induced phosphorylation of cytoplasmic p65. IL-1β also induced p65 phosphorylation in the nuclear fraction in a time dependent manner, and PDGF-bb and IGF-1 blocked IL-1β-induced translocation of p65 to the nucleus (Fig. 8A–B). Interestingly, co-treatment of the chondrocytes with a combination of the two growth factors decreased the levels of these proteins more than each agent by itself (Fig. 8C).

### Effect of IGF-1 or/and PDGF-bb on IL-1β-induced acetylation of NF-κB in chondrocytes in monolayer cultures

**Figure 9:**
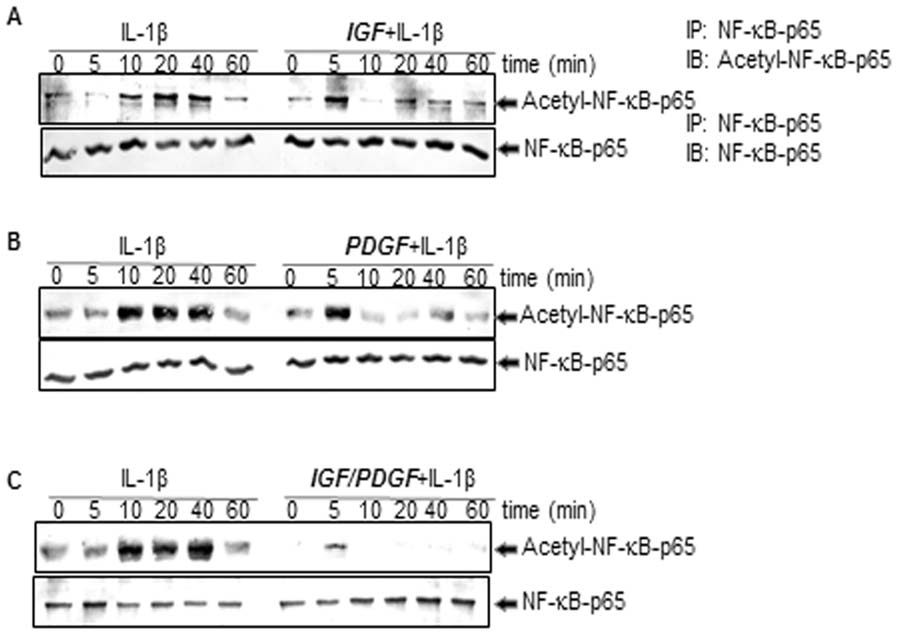
A–C: Effects of IGF-1 or/and PDGF-bb on the IL-1β-induced p65 acetylation in chondrocytes in monolayer cultures. Primary chondrocytes were either stimulated with 10 ng/ml IL-1β, pre-stimulated with 10 ng/ml IGF-1, 10 ng/ml PDGF-bb or a combination of both growth factors (5 ng/ml each) for 12 h and treated with 10 ng/ml IL-1β for the indicated times. Whole-cell extracts were prepared and immunoprecipitated with an anti-p65 antibody. Western blot analysis was then performed with an anti-acetyl-lysine antibody or with an anti-p65 antibody. The results shown are representative of three independent experiments.

Acetylation of p65 plays an essential role in IκBα-mediated activation of NF-κB transcriptional activity [32], therefore we investigated the effect of PDGF-bb or/and IGF-1 on the induction of p65 acetylation by IL-1β. The chondrocytes were pre-treated with PDGF-bb or/and IGF-1 for 4 h and then exposed to IL-1β for the indicated times. Whole-cell extracts were examined and immunoprecipitated with anti-p65 antibody, and western blot analysis was performed using anti-acetyl lysine antibody. As shown in Fig. 9, IL-1β induced acetylation of p65 in a time dependent manner. In contrast to this, pre-treatment of chondrocytes with the PDGF-bb or/and IGF-1 followed by stimulation with IL-1β resulted in an inhibition of cytokine-induced effects on the acetylation of p65 (Fig. 9A–C). Interestingly, co-treatment of the chondrocytes with combinations of the two growth factors completely suppressed the IL-1β-induced acetylation of p65 (Fig. 9C).

### Effect of IGF-1 or/and PDGF-bb on IL-1β-induced activation of IKK in chondrocytes in monolayer cultures

**Figure 10:**
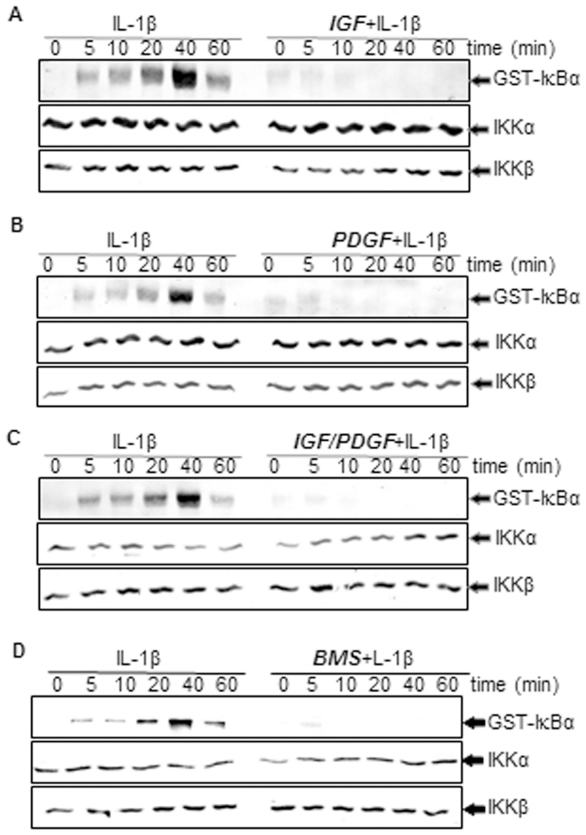
A–D: Effects of IGF-1 or/and PDGF-bb or IKK-inhibitor (BMS) on the IL-1β-induced IKK activation in chondrocytes in monolayer cultures. Primary chondrocytes were either stimulated with 10 ng/ml IL-1β, pre-stimulated with 10 ng/ml IGF-1, 10 ng/ml PDGF-bb, a combination of both growth factors (5 ng/ml each) or 5 µM BMS-345541 (BMS) for 12 h and treated with 10 ng/ml IL-1β for the indicated times. Whole-cell extracts were immunoprecipitated with an antibody against IκB kinase (IKK)and then analyzed by an immune complex kinase assay as described in Materials and Methods. To examine the effect of *IGF-1 or/and PDGF-bb or* BMS on the level of activation of IKK proteins, whole-cell extracts were fractionated (500 ng protein per lane) on SDS–PAGE and examined by western blot analysis using anti-IKK-α and anti-IKK-β antibodies. The results shown are representative of three independent experiments.

IKK is required for cytokine-induced phosphorylation of IκBα [31]. We further evaluated the effect of PDGF-bb or/and IGF-1 on IL-1β-induced IKK activation, which is required for IL-1β-induced phosphorylation of IκBα. The results from the immune complex kinase assay showed that IL-1β induced the activation of IKK in a time dependent manner. In contrast, pre-treatment of chondrocytes with the PDGF-bb or IGF-1 followed by stimulation with IL-1β resulted in an inhibition of cytokine-induced effects on the activation of IKK (Fig. 10A–B). Co-treatment of the chondrocytes with combinations of the two growth factors completely suppressed IL-1β-induced activation of IKK more than each agent by itself (Fig. 10C). To determine whether BMS-345541, the specific inhibitor of IKK [29] can inhibit the NF-κB pathway like PDGF-bb or/and IGF-1, we analysed the effect of BMS on IL-1β-induced IKK activation, which is required for IL-1β-induced phosphorylation of IκBα. Immune complex kinase assays showed that IL-1β induced the activation of IKK in a time dependent manner. In contrast, pre-treatment of chondrocytes with BMS followed by stimulation with IL-1β resulted in an inhibition of cytokine-induced effects on the activation of IKK (Fig. 10D). IL-1β, BMS, *PDGF-bb or/and IGF-1* had no direct effect on the expression of IKK-α or IKK-β proteins (Fig. 10).

### Effect of IGF-1 or/and PDGF-bb on IL-1β-induced activation of Akt in chondrocytes in monolayer cultures

**Figure 11:**
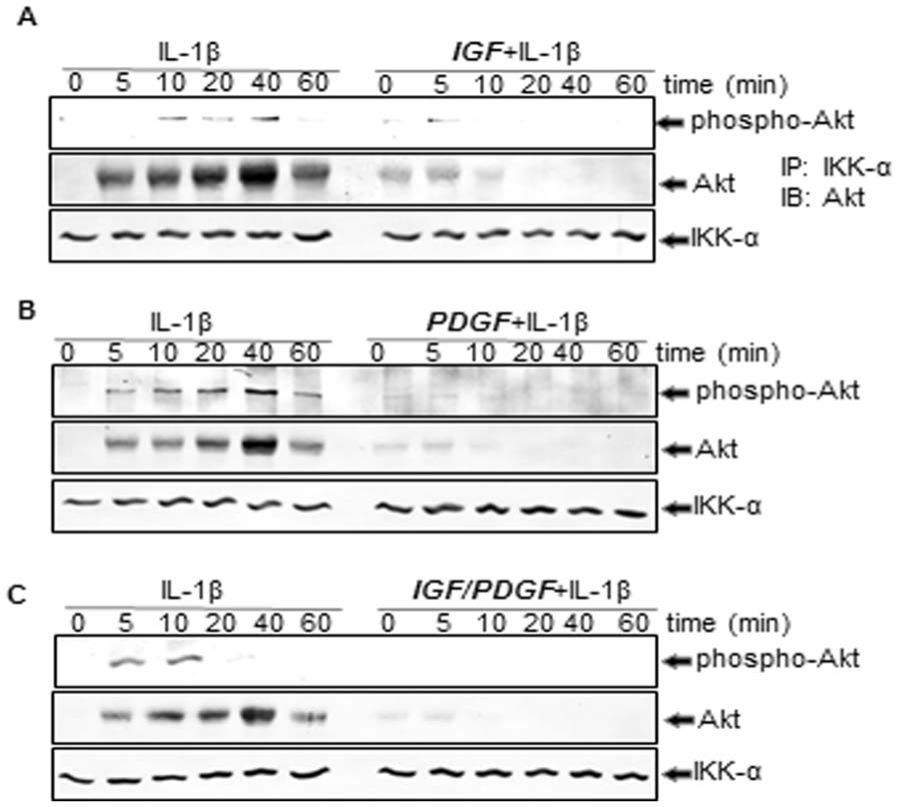
A–C: Effects of IGF-1 or/and PDGF-bb on the IL-1β-induced Akt activation in chondrocytes in monolayer cultures. Primary chondrocytes were either stimulated with 10 ng/ml IL-1β, pre-stimulated with 10 ng/ml IGF-1, 10 ng/ml PDGF-bb or a combination of both growth factors (5 ng/ml each) for 12 h and treated with 10 ng/ml IL-1β for the indicated times. Whole cell extracts were immunoprecipitated with anti-IKK-α antibody followed by western blot analysis using anti-Akt, anti-phospho-specific Akt and anti-IKK-α antibodies. The results shown are representative of three independent experiments.

Activation of NF-κB requires up-stream kinases including Akt [33], therefore we examined the effect of growth factors on Akt activation. Cells were treated with PDGF-bb or/and IGF-1 and then with IL-1β, and cell extracts were prepared followed by western blot analysis using anti-Akt or anti-phospho specific-Akt antibodies. The results clearly show that IL-1β induced Akt activation in a time-dependent manner; PDGF-bb or IGF-1 inhibited this process (Fig. 11 A–B). Co-treatment of the chondrocytes with combinations of the two growth factors decreased the levels of this protein more than each agent by itself (Fig. 11 C). Further, we wanted to know whether these growth factors impair the association of Akt-protein with IKK-protein. Therefore, extracts were prepared from IL-1β-treated cells followed by immunoprecipitation with anti-IKK-α antibody and western blot analysis using anti-Akt antibody. IL-1β induced association between IKK and Akt in a time-dependent manner and PDGF-bb or/and IGF-1 suppressed that (Fig. 11 A–B). Co-treatment of the chondrocytes with combinations of the two growth factors decreased the levels of this association between Akt and IKK protein more than each agent by itself (Fig. 11 C).

### Effect of c-Src inhibitor (PP1) and IGF-1 or/and PDGF-bb on IL-1β-induced c-Src phosphorylation in chondrocytes

**Figure 12:**
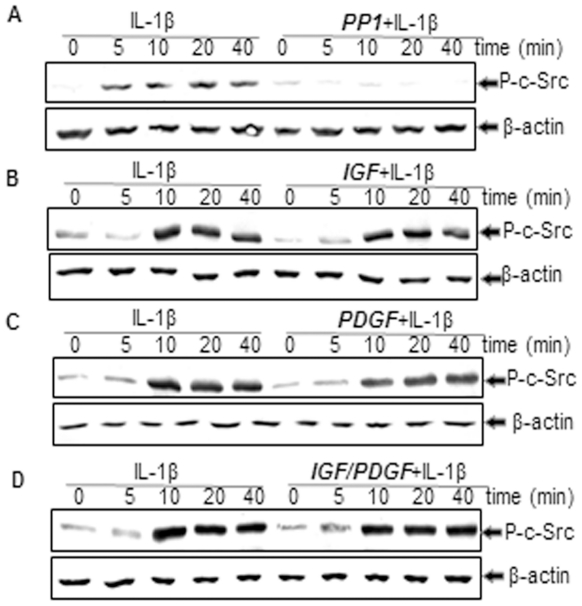
A–D: Effects of PP1 and IGF-1 or/and PDGF-bb on IL-1β-induced c-Src phosphorylation in chondrocytes in monolayer cultures. Primary chondrocytes were either stimulated with 10 ng/ml IL-1β, pre-stimulated with 10 ng/ml IGF-1, 10 ng/ml PDGF-bb or a combination of both growth factors (5 ng/ml each) for 12 h, or with PP1 (10 µM) for 1 h and treated with 10 ng/ml IL-1β for the indicated times. The cell lysates were subjected to 10% SDS-PAGE (500 ng protein per lane), transferred to nitrocellulose membranes and then probed using anti-phospho-Src and anti-β-actin (as an indicator of protein loading in each lane) antibodies. Identical results were obtained in three independent experiments.

To examine whether c-Src phosphorylation was necessary for the IL-1β-induced NF-κB induction, activation of this kinase was determined by western blotting with an antibody specific for the phosphorylated c-Src. As shown in Fig. 12A, IL-1β stimulated phosphorylation of c-Src in chondrocytes and reached a maximum within 5 to 10 min. This IL-1β-stimulated c-Src phosphorylation was inhibited by pre-treatment with inhibitors of c-Src (PP1), but not by IGF or/and PDGF-bb (Fig. 12B-D). These results indicate that IL-1β-stimulated c-Src phosphorylation was not mediated through IGF or/and PDGF. Further, these results suggest a link between activation of c-Src and induction of NF-κB activation by IL-1β in chondrocytes.

### Effect of Src-, PI-3K-, AKT-inhibitors and IGF-1 or/and PDGF-bb on IL-1β-induced phosphorylation of NF-κB in chondrocytes

**Figure 13:**
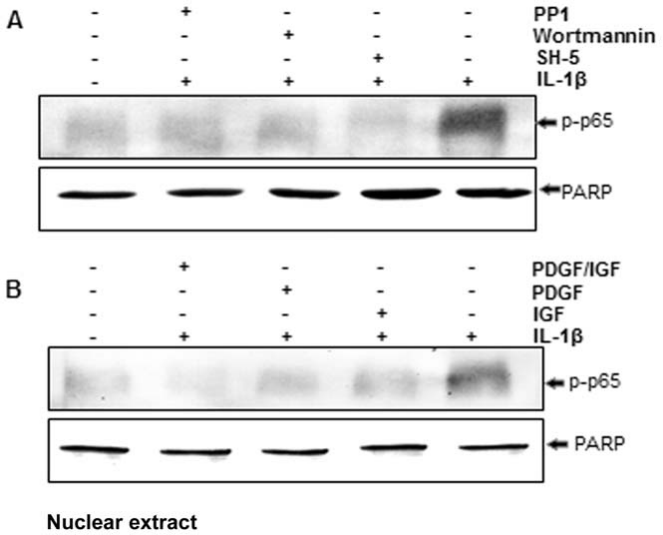
A–B: Effects of Src-, PI-3K-, AKT-inhibitors and IGF-1 or/and PDGF-bb on IL-1β-stimulated phosphorylation of NF-κB in chondrocytes. Primary chondrocytes were either stimulated with 10 ng/ml IL-1β, pre-stimulated with PP1 (10 µM), wortmannin (20 nM) and SH-5 (10 µM) for 1 h (A), or with 10 ng/ml IGF-1, 10 ng/ml PDGF-bb or a combination of both growth factors (5 ng/ml each) for 12 h (B) and then incubated with IL-1β for 30 min. Nuclear extracts were subjected to 10% SDS-PAGE (500 ng protein per lane), transferred to nitrocellulose membranes and then probed using an antiserum reactive with an anti-phospho-p65 or anti-PARP polyclonal antibody (housekeeping control). Similar results were obtained in three independent experiments.

To investigate the role of the c-Src/PI-3K/Akt signaling pathway in regulating of IL-1β-mediated NF-κB activation, the level of NF-κB protein was analyzed using selective kinase inhibitors. Chondrocytes were pre-treated with inhibitors of c-Src (PP1), PI-3K (wortmannin) and Akt (SH-5), respectively for 1 h, and then co-treated with 10 ng/ml IL-1β for 1 h (Fig. 13A). In parallel with the inhibition of NF-κB activation, chondrocytes were pre-treated with IGF-1 (10 ng/ml) or PDGF-bb (10 ng/ml) or a combination of both growth factors (each 5 ng/ml) for 4 h, and then exposed to 10 ng/ml IL-1β for 1 h (Fig. 13B). As shown in Fig. 13A–B, the activation of NF-κB in chondrocytes was significantly increased by incubation with IL-1β. However, the specific inhibitors for c-Src, PI-3K and Akt blocked IL-1β-stimulated NF-κB. In addition, IL-1β-stimulated NF-κB activation was also significantly inhibited by pre-incubation with IGF-1 or/and PDGF-bb. These results further suggest that IGF-1 or/and PDGF-bb play an important role in down-regulation of the IL-1β-induced NF-κB activation in chondrocytes, at least in part through inhibition of the c-Src/PI3K/Akt signaling pathway.

## Discussion

In this study we examined whether the anti-inflammatory and anti-apoptotic effects of IGF-1 or/and PDGF-bb in primary chondrocytes are mediated through similar signalling mechanisms and whether combining these two growth factors exerts synergistic effects on IL-1β-mediated cellular responses, NF-κB mediated signal transduction pathways and the regulation of NF-κB regulated gene expression. Our results demonstrated for the first time evidence that in chondrocytes, IKK-inhibitor (BMS), IGF-1 or/and PDGF-bb can suppress IL-1β-induced NF-κB activation and apoptosis, and this may be mediated through c-Src dependent suppression of PI-3K/Akt, revealed by using pharmacological inhibitors PP1, wortmannin and SH-5, respectively.

In several previous studies, we have demonstrated that IL-1β induces down regulation of chondrocyte ECM molecules and apoptosis through activation of NF-κB dependent pathways and growth factors can block these effects [14], [25], [34], [35]. Therefore, in this study, we examined whether alternative signaling molecule(s) to downstream pathway of IL-1β, such as c-Src/PI-3K/Akt activation pathway, are involved in IL-1β-induced NF-κB activation and cell apoptosis in chondrocytes. Using monolayer cultured chondrocytes we found that IGF-1 or/and PDGF-bb or BMS inhibit IL-1β-induced NF-κB activation through the suppression of IκBα phosphorylation, IκBα degradation, IκBα kinase activity and NF-κB–dependent gene products involved in inflammation (COX-2, MMPs) and activation of caspase-3. Moreover, inhibition of NF-κB activation by IGF-1 or/and PDGF-bb or BMS was correlated with suppression of p65 phosphorylation, p65 nuclear translocation and p65 acetylation.

Previous work from our group has shown that growth factors (IGF-1 or/and TGF-β1) exert additive anabolic and anti-inflammatory effects on IL-1β signalling pathways in chondrocytes *in vitro* [12], [26]. In the present study, we demonstrated that growth factors (IGF-1 or/and PDGF-bb) exert their anabolic and anti-inflammatory effects on the maintenance of the chondrogenic potential in chondrocytes *in vitro* by inhibiting of NF-κB pathway. Moreover, several investigators have reported that many pro-inflammatory effects of IL-1β and TNF-α are regulated by stimulation of the ubiquitous transcription factor NF-κB [36], [37], [38], which plays a crucial role in OA, leading to cartilage destruction and articular damage [39]. Suppression of NF-κB by IGF-1 or/and PDGF-bb or BMS modulates the activation of NF-κB-regulated gene products (i.e. COX-2, MMP-9 and MMP-13, caspase-3 cleavage), which partly explains the anti-apoptotic and anti-inflammatory effects of IGF-1 or/and PDGF-bb in chondrocytes.

In this study we show that IL-1β-induces down-regulation of collagen type II, cartilage-specific proteoglycans, β1-integrin and the expression of MAPKinase specific proteins. This process is accompanied by inhibition of the SOX-9 protein expression in chondrocytes, in agreement with previous studies of articular chondrocytes from our and other laboratories [12], [34], [40]. Moreover, SOX-9 is required for expression of cartilage-specific ECM molecules in cartilage [41], [42]. Interestingly, in the present study, the down-regulation of the mentioned proteins was inhibited by pre-treating the cells with a specific IKK-inhibitor (BMS) or IGF-1 or/and PDGF-bb followed by stimulation with IL-1β. Further, co-treatment of the chondrocytes with combinations of the both growth factors increased the levels of these proteins more than each agent by itself. Moreover, as it has been previously demonstrated by our group, activation of β1-integrin and adaptor protein Shc are key events for stimulation of MAPKinase intracellular signaling pathway [20], [43], which up regulates SOX-9 expression in chondrocytes *in vitro* [12]. In agreement with previous reports from other laboratories, we have revealed that pro-inflammatory cytokines could reduce transcription factor SOX-9 expression by a IKK-NF-κB-dependent pathway, post-transcriptional mechanism in chondrocytes [44], [45], suggesting a collaboration between growth factors in a common/shared biochemical signaling pathway.

Activation of growth factor receptor results in regulation of gene expression involved in cell proliferation, survival, migration or anti-inflammatory responses [46]. We found that IGF-1 and PDGF-bb suppressed the IL-1β-induced NF-κB activation. How IGF-1 and PDGF-bb suppress IL-1β-induced NF-κB activation in chondrocytes is not fully understood. In response to IL-1β, NF-κB activation proceeds sequentially through activation of IKK, phosphorylation and ubiquitination of IκB-α, and finally degradation of IκB-α and the release of NF-κB [31]. How IGF-1 or/and PDGF-bb inhibits IKK activation was also investigated. The multiple signaling steps that lead to NF-κB activation are not fully understood. Further, protein kinase B (Akt) has been reported to activate IKK [33]. Our results show that IGF-1 or/and PDGF-bb suppressed IL-1β-induced Akt activation as well as Akt-IKK association. These results indicate that IGF-1 and PDGF-bb may inhibit IKK activation through suppression of Akt activation. Moreover, it has been shown that IL-1β activates the up-stream kinase c-Src which in turn stimulates the downstream PI-3K/Akt signaling pathway [47]. Thus, we examined whether PI-3K plays a role in IL-1β-induced NF-κB activation and whether wortmannin inhibits PI-3K pathway in chondrocytes, since it was not reported before. Our results show that wortmannin like IGF-1 and PDGF-bb inhibited NF-κB activation in chondrocytes. In fact, it has been shown that NF-κB activation requires a PI-3K/Akt signaling pathway [33], [48], [49]. The results obtained in this study with IL-1 β-induced NF-κB activation are consistent with these reports. Our results also indicate that the activation of NF-κB by IL-1β is blocked by SH-5 and PP1, specific inhibitors of protein kinase B (Akt) and c-Src. In fact, it has been reported that Src and Akt could play a synergistic functional cooperation with PI-3K [33], [47].

We found further that IL-1β induces NF-κB activation in a concentration-dependent manner and c-Src was required for the NF-κB activity induced by IL-1β. This activation was inhibited by a specific c-Src inhibitor (PP1) but not by IGF-1 or/and PDGF-bb. Further, the data showed that activation of c-Src by IL-1β was linked to the PI-3K/Akt cascade leading to NF-κB induction and activity in chondrocytes. These results suggest that in chondrocytes, IL-1β-induced NF-κB activation and activity is mediated, at least in part through a c-Src/PI-3k/Akt component and this signaling pathway was inhibited by receptor tyrosine kinases, including the insulin-like growth factor-1 receptor and the platelet-derived growth factor. These findings further confirmed that IL-1β-induced NF-κB activation was mediated through either PI-3K/Akt-dependent pathway or c-Src-dependent activation of the PI-3K/Akt pathways.

The precise nature of the connection between IL-1β signaling and downstream kinases is not well established in chondrocytes. It is known that PI-3K/Akt signaling pathway is involved in IL-1β-mediated signalling events and this pathway is activated originating from cell surface receptors, such as PDGFR and other growth factors receptors in various cell types [50]. In fact, several reports have shown that Akt and PI-3K/Akt phosphorylation is required for pro-inflammatory cytokine-induced NF-κB activation in various cell types [33], [47], [49]. Our results were consistent with these studies demonstrating that Akt is an important protein in this cascade for NF-κB activation and translocation from cytosol into nucleus through targeting IKKs or phosphorylation of p65 [51] in response to IL-1β and Akt is a downstream target for inhibition effects of IGF-1 or/and PDGF-bb in chondrocytes. Several lines of evidence have reported that IL-1β/IL-1βR induced phosphorylation of tyrosine residues of c-Src [47], [51], [52], but little is known about the mechanisms underlying IL-1β-stimulated NF-κB activation mediated through c-Src-dependent pathway in chondrocytes. Our results also showed that IL-1β-enhanced NF-κB (p65) phosphorylation and translocation was significantly attenuated by pre-treatment with the inhibitors of Src, PI3K, Akt and the both growth factors, indicating that activation of growth factor receptors and Src/PI-3K/Akt pathway play an important role in modulating IL-1β-mediated NF-κB (p65) activation. Our results demonstrate here that a specific IKK inhibitor (BMS) suppresses the destructive effects of IL-1β like IGF-1/PDGF-bb. Moreover, it has been shown in other cell types, that Akt directly phosphorylates IKKs leading to the activation of NF-κB, independent of mitogen-activated protein kinase kinase-1 and NF-κB inducing kinase [33]. IKK is, at least in part, the kinase that growth factor signalling directly interferes with in the Src/PI3K/Akt pathway. This pathway thus allows growth factors such as PDGF or/and IGF-1 to inhibit NF-κB activation.

In addition, these results suggest that growth factors added to or mixed in a cell pellet or 3-D construct can induce both anabolic mediators and inhibit possible sources of inflammation, making these growth factors ideal compounds for use in cells used in autologous chondrocytes transplantation.
